# A New Approach to Evaluate the Bactericidal Activity of Different Antiseptic Ophthalmic Preparations Used as Surgical Prophylaxis

**DOI:** 10.3390/antibiotics13111051

**Published:** 2024-11-06

**Authors:** Sara Caldrer, Niccolò Deotto, Marco G. Prato, Natasha Gianesini, Milena Bernardi, Pierantonio Orza, Federico G. Gobbi, Grazia Pertile, Chiara Piubelli

**Affiliations:** 1 Department of Infectious—Tropical Diseases and Microbiology, IRCCS Sacro Cuore—Don Calabria Hospital, 37024 Negrar di Valpolicella, Italychiara.piubelli@sacrocuore.it (C.P.); 2 Department of Ophthalmology, IRCCS Sacro Cuore Don Calabria Hospital, Via Don A. Sempreboni, 5, 37024 Negrar di Valpolicella, Italy; 3 Department of Clinical and Experimental Sciences, University of Brescia, 25121 Brescia, Italy

**Keywords:** infectious disease, ocular infection, bactericidal activity, ophthalmic preparation, surgical prophylaxis

## Abstract

**Background**: A survey conducted by the European Observatory on Cataract Surgery showed high heterogeneity in the use of antiseptics both preoperatively and in the operating room, highlighting the absence of a global consensus regarding ocular infection prophylaxis in cataract surgery. **Methods**: The antibacterial activity of seven antiseptic ophthalmic formulations (AOFs) registered as medical devices and the two most common disinfectants were evaluated in vitro against five bacterial species. The viability of bacterial strains after exposure to the antiseptic was evaluated with different techniques: the in vitro Minimum Inhibitory Concentration and the subsequent Minimum Bactericidal Concentration, performed on liquid and solid culture medium. Furthermore, a real-time assessment of bacterial viability was conducted using double staining for live/dead bacterial cells by fluorimetric assay. This evaluation was performed on both the time-killing curve and the tear dilution effect test. **Results**: We observed a high variability across the different AOFs in terms of inhibitory/bactericidal concentration and timing on Gram-positive and Gram-negative bacterial classes. The results indicated that among the tested AOFs, Visuprime, Iodim, and Oftasteril were the most rapid and effective for ocular surface disinfection against the tested bacterial species. **Conclusions**: The obtained results can support the clinician’s choice of the most suitable AOF for the prevention and treatment of ophthalmic infections associated with surgery.

## 1. Introduction

The reduction of bacterial load on the periocular skin and ocular conjunctiva before surgical procedures is of paramount importance to avoid the development of surgery-related ocular infections [1,2]. Despite the application of surgical prophylaxis, such as disinfection of the surgical site, eyelids, and conjunctiva, the incidence of endophthalmitis has increased over the past few decades [3,4,5]. Several studies indicate that the bacteria residing in the lids, tear film, and adnexa are the main agents responsible for infection. It is also worth noting that the most common microorganisms implicated in postoperative endophthalmitis are coagulase-negative staphylococci (CNS), especially *Staphylococcus epidermidis* and *Staphylococcus aureus* [6,7]. Kowalski et al. in 2019 fully described the distribution of infectious agents for keratitis, endophthalmitis, and conjunctivitis between 2004 and 2018, drawing a scale representing the prevalence of infectious agents in cases of infection [8]. These organisms are thought to be the most commonly present in the microbiome of patients’ eyelid and conjunctiva [9]. Molecular epidemiology techniques revealed that the microbes isolated from the vitreous were, in over 80% of cases, identical to the conjunctival microorganisms isolated from the patient’s eyelid [6].

In this scenario, preoperative prophylaxis plays a key role in the prevention of postoperative endophthalmitis. A survey conducted by the European Observatory of Cataract Surgery revealed a considerable degree of heterogeneity between European countries in the use of antiseptics, both before and during the surgical procedure [3,4,10]. This led to the conclusion that there is no global consensus on endophthalmitis prophylaxis for cataract surgery [4].

In 1984, Apt et al. first demonstrated the effect of povidone–iodine (PVI) on the conjunctiva, with about 90% reduction in ocular surface microorganisms [11], leading to PVI being considered the most effective antiseptic for reducing the incidence of postoperative endophthalmitis [11,12]. More recently, in 2013, the European Society of Cataract and Refractive Surgeons (ESCRS) published the “Guidelines for Prevention and Treatment of Endophthalmitis Following Cataract Surgery”, which unequivocally highlighted the clinical benefit of the intra-cameral injection of cefuroxime at the end of cataract surgery, citing a fivefold reduction in the incidence of endophthalmitis [13].

To avoid the development of post-surgical infections, it is necessary to implement a series of effective measures, designed to prevent pre-surgical contamination of the ocular surface and to limit the inappropriate use of antibiotics [14,15,16]. In recent years, the discovery of new molecules that are well-tolerated by biological tissues and capable of performing effective antibacterial actions, without the risk of the development of antibiotic resistance, represents an effective strategy for ophthalmologists. Therefore, the field of infection prevention in the operating room has expanded to encompass the use of novel Antiseptic Ophthalmic Formulations (AOFs) such as Iodim, Ozodrop, Corneial MED, Keratosept, Dropsept, Visuprime, and Oftasecur. 

Before being approved for use, antibiotic drugs are subjected to antimicrobial susceptibility tests (AST), in order to ascertain the efficacy of the antibiotic treatment against different bacterial species [17,18,19]. Since AOFs are considered only as medical devices, this procedure is not requested, and each distributing company performs limited studies to support the efficacy evidence. Thus, there aren’t clear and strong indications for the use of these eye drops, based on the infection’s etiology. In the literature, we found two main study types about ocular antiseptics evaluation: one type exploring the minimum effective concentration (usually involving EUCAST-based microdilution) and the other assessing the kinetic (usually sampling a mixed solution of microbes and antimicrobial drug at different time points for vitality evaluation) [18,20]. These studies are based on tests using only viable and culturable cells and are prone to errors, due to factors involved in bacterial growth on agar plates. Moreover, due to the very short contact time of AOFs with the ocular surface, these tests are not able to recapitulate the in-vivo conditions [21]. Time-kill curves (TKC) can follow microbial killing and growth as a function of both time and antibiotic concentration, to mimic an in vivo concentration profile. Consequently, AOFs should also be tested by optimal AST, which could accurately reflect the effective ocular situation, possibly reducing time-consuming steps.

An alternative approach to culture-based detection methods is the assessment of cell viability indicators using fluorescent dyes, such as SYTO 9 and propidium iodide (PI). These dyes can differentiate live and dead cells based on membrane integrity, which is a proxy for cell viability. The signals from the dyes are typically measured using fluorescence microscopy, fluorescence-based microplate readers (FMR), or flow cytometry (FCM) [22,23,24]. Compared to microscopy, FCM allows visualization of the gradual loss of membrane integrity and is characterized by superior ease of use [25]. Otherwise, FMR can be automated and performed in a high-throughput manner [26]. However, the accuracy of the microplate reader and FCM measurements depends on the sensitivity of the instrument and requires trained technicians. Previous works demonstrated the usefulness of these techniques based on dual staining in the rapid and effective identification of live and dead cell suspensions of different bacterial species [21,22,23,24,27,28,29].

In this study, we assessed the in vitro antimicrobial activity of several AOFs against five bacterial species independently, which are the most representative pathogens causing ocular infections, with the main objective of supporting the clinician’s choice of the most suitable AOF for the prevention and treatment of surgery-associated ophthalmic infections. Both the minimum inhibitory concentration (MIC) and the minimum bactericidal concentration (MBC) were performed, as well as the time-killing curve through either conventional or fluorescence-based approaches. Moreover, to reproduce the in vivo dynamics of AOF action in the ocular surface microenvironment in the presence of tear washing, we designed a new test to resemble the rapid and considerable dilution of the antiseptic concentration due to the tear flow, called the Tear Flow–Mimicking Curve (TFMC) [30,31].

## 2. Material and Methods

### 2.1. Pathogens

The antibacterial activity of each AOF was evaluated in vitro against six different bacterial species: *Staphylococcus aureus* (ATCC 29213), *Staphylococcus epidermidis* (ATCC 12228), *Streptococcus pneumoniae* (ATCC 49619), *Pseudomonas aeruginosa* (ATCC 9027), and *Serratia marcescens* (ATCC 13880). Microorganisms were obtained from ATCC (American Type Culture Collection; Manassas, VA, USA).

### 2.2. Ophthalmic Formulations

The AOFs used in the study and their respective compositions are listed in Table 1. For each formulation, the following serial dilutions were considered in the MIC and MBC assays: 75%, 60%, 45%, 30%, 15%, 5%, and 1% of the starting commercial preparation. To mimic the “tears flow dilution effect” in normal conditions, in the TFMC experiment the AOFs concentrations were reduced by 15% every minute as previously described [30,31]. Starting from a minimal dilution of 75%, after 8 min we obtained a final AOF dilution of 20.4%. For all the following tests, the maximum antibacterial efficacy was measured using an aqueous solution of Chlorhexidine 0.05% as efficacy control (CLX), and bacteria inoculum without any antiseptic solution as non-treated control (NT) and growth check. Despite Oftasteril being an operating room eye disinfectant and not an eye drops product, we used it in order to evaluate its effectiveness as for AOFs.

### 2.3. The Study Design

**Figure 1:**
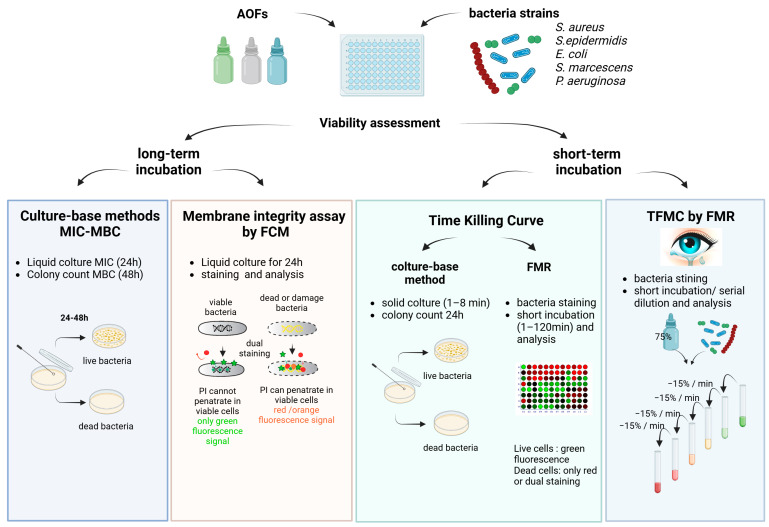
Study design representing the different techniques employed, classified according to the time of incubation.

The viability of bacterial species after exposure to AOFs was evaluated with different techniques, depending on the incubation time, as shown in Figure 1. The long-term assay encompasses both the MIC and the MBC, as well as the membrane integrity assay conducted after 24 h of incubation, performed by FCM. Short-term assays were conducted on both the TKC, through either conventional and fluorescence-based approaches, and the TFMC assay through the FMR technique.

### 2.4. Minimum Inhibitory Concentrations and Minimum Bactericidal Concentrations (Long-Term Incubation)

To determine the in vitro MIC of each ophthalmic solution, micro-broth dilution assays were performed in agreement with the Clinical and Laboratory Standards Institute Guidelines [32]. Briefly, from a suspension at 600 Colony Forming Unit per mL (CFU/mL) in fresh cation-adjusted Mueller–Hinton Broth (MHB, Thermo Scientific™ Fisher Scientific Italia, 20054 Segrate (MI), Italy), 50 µL was inoculated in flat-bottom 96-well polystyrene microtiter plates containing serial dilutions of the AOF to reach the bacterial final concentration of 150 CFU/mL per well [33]. In a 96-well sterile microtiter plate, 15-fold serial dilutions from 75% to 1% of each ocular formulation (listed in Table 1) were placed by diluting AOF in MHB. In the same plate, CLX and NT controls were used. A sterile control for the broth was provided as well. Into each well of the plate, 150 µL of ophthalmic solution and 50 µL of bacteria were mixed and incubated at a temperature of 37 °C for 24 h (±2 h).

After incubation, the MIC of each AOF was calculated as the lowest dilution of the drug causing the growth inhibition, visible by the absence of any clot at the bottom or turbidity of the broth. The MBC was determined by plating, on a blood agar plate, 10 µL of suspension from the wells corresponding to the MIC value and the next two dilutions for all the ophthalmic formulations. The plates were incubated at 37 °C for an additional 24 h. The MBC was identified as the lowest dilution that prevents any microbial growth on the agar plate. Growth was evaluated by manually counting the colonies on the plate and reporting the number (from 0 to >1000 colonies) according to the initial plated volume (CFU/mL). Triplicates of each assay were performed on different days and data was shown as mean, as described in Supplementary Table S1. All graphs were generated using the GraphPad software Prism 9.0 (San Diego, CA, USA).

### 2.5. Viability Assessments Based on Membrane Integrity by FCM (Long-Term Incubation)

After the MIC measurements, cell viability was also evaluated by FCM with double staining with the LIVE/DEAD BacLight Bacterial Viability Kit (Thermo-fisher; Invitrogen, Carlsbad, CA, USA). It consists of two nucleic acid stains. Green fluorescent SYTO 9 is cell-permeable and freely enters all tested bacteria, either live or dead. In contrast, red fluorescent PI can only enter cells with a compromised membrane. Therefore, the measured fluorescence with SYTO 9/PI dual staining is directly related to bacteria membrane integrity [34]. The test was performed according to the manufacturer’s instructions with minor modifications. In our final setup, 50 μL of 24 h-treated bacteria samples obtained after the MIC evaluation, were harvested in a 96-well plate and then stained with a mixture of SYTO 9 and PI (3.5 μL each/in 5 mL) and incubated for 15 min in the dark at room temperature. Subsequently, the stained samples were analysed by Cytoflex (BD Biosciences, Milan, Italy) with FSC-H and SSC-H thresholds at 4000 and 1000, respectively, and a stopping rule set on 30.000 events. Our FCM protocol was optimized to monitor the viability of *S. epidermidis*, *S. aureus*, *S. pneumoniae*, *P. aeruginosa*, and *S. marcescens*. Live cells were detected by the green FL1 channel (excitation at 488 nm, emission filter 530), and dead cells or cellular debris were detected by the red FL3 channel (excitation at 488 nm, emission filter 630) as described in Supplementary Figure S1. Mean Fluorescence intensity (MFI), and the relative frequency (%) of the different cell populations (live/damaged/dead) were finally measured using Kaluza software v2.1 (Beckman Coulter, Brea, CA, USA).

### 2.6. The Conventional TKC (Short-Term Incubation)

The conventional colony counting-based method was used for this study [32] but has been adapted to mimic the real amount of bacteria and AOFs on the infected eye surface [33,35]. Briefly, 50 µL of the microbial inoculum was exposed to 150 µL (3 drops) of the different AOFs at the minimal dilution of use (75%) for 1, 3, 5, and 8 min. At every time point, 50 µL was seeded on Agar Chocolate plates (or Blood Agar plates for *S. pneumoniae*) for 24 h at 37 °C and then evaluated by manually counting colonies. CLX and NT controls were used. The reduction in colony count was plotted against time.

### 2.7. Viability Assessments Based on Membrane Integrity by FMR and TFMC Assays (Short-Term Incubation)

Cell viability was evaluated using the LIVE/DEAD BacLight Bacterial Viability Kit (Thermo-fisher, Waltham, MA, USA). For both assays, 5 mL of each bacteria species were stained with the mixture of SYTO 9 and PI (3.5 μL each) and incubated for 15 min in the dark at room temperature to allow dye penetration and linking with live cells. Subsequently, 25 μL of dye-bacterial suspension (final concentration of 150 CFU/well) was harvested in each well of a black 96-well plate (Thermo Scientific, light-tight, flat bottom, non-sterile). Then, 75 μL of each AOF (75% dilution) was added to each well.

For the TKC experiments, the plate was then read at different time points for 120 min of total incubation, using a Varioskan™ LUX multimode microplate reader (Thermo Scientific™) for the green (530 nm) and red (630 nm) fluorescence emission intensity measurement. A CLX 0.05% solution was used as efficacy control. A 20% deviation from the standard CLX treatment was employed as a reference point for the evaluation of the AOFs’ efficacy.

For the TFMC, the plate was immediately read after 1 min of incubation. Subsequently, every minute, an amount of 15 µL of bacteria–AOF mixture (15% of total volume) was substituted with 15 µL of physiological water, as described in Figure 1. This procedure was adopted to mimic the physiological “tears flow dilution effect” on the AOFs and bacteria concentration on the ocular surface. In this case, the experimental control consisted of a solution of stained living bacteria to evaluate the decrease in fluorescence due to the reduction of amount of stained bacteria.

### 2.8. Data Analysis: AFU and N0/N

We analysed the data by considering the fluorescence intensity of the stained bacterial suspensions at the emission of 530 nm (SYTO9) and the emission of 630 nm (PI). For FCM evaluation, we calculated the Active Fluorescence Unit (AFU), as described by Mattio et al. [21], which was directly proportional to the live bacteria amount (green fluorescence_SYTO 9).

Meanwhile, for the FMR evaluation with the microplate reader (Varioskan LUX multimode microplate reader), we recorded both fluorescence peaks (SYTO9 and PI) [36]. Due to the previously described issue in the dye behaviour [28,37], we performed two kinds of corrections. First, we subtracted the auto-fluorescence intensity due to the AOFs’ own colour (like brown iodiopovidone and micellar Ozodrop). Second, we subtracted the PI fluorescence in the SYTO 9 emission during every time point and then, to compare the data obtained from the different bacterial species, we normalized all the data in the kinetic test for the initial values by calculating a ratio N/N0 (AFU at time N/AFU at the initial time), as described in Supplementary Tables S3 and S4. All values were compared to the CLX or NT referral control.

## 3. Results

### 3.1. Inhibitory Effect and Antibacterial Efficacy of AOFs Against Cultured Bacteria

**Figure 2:**
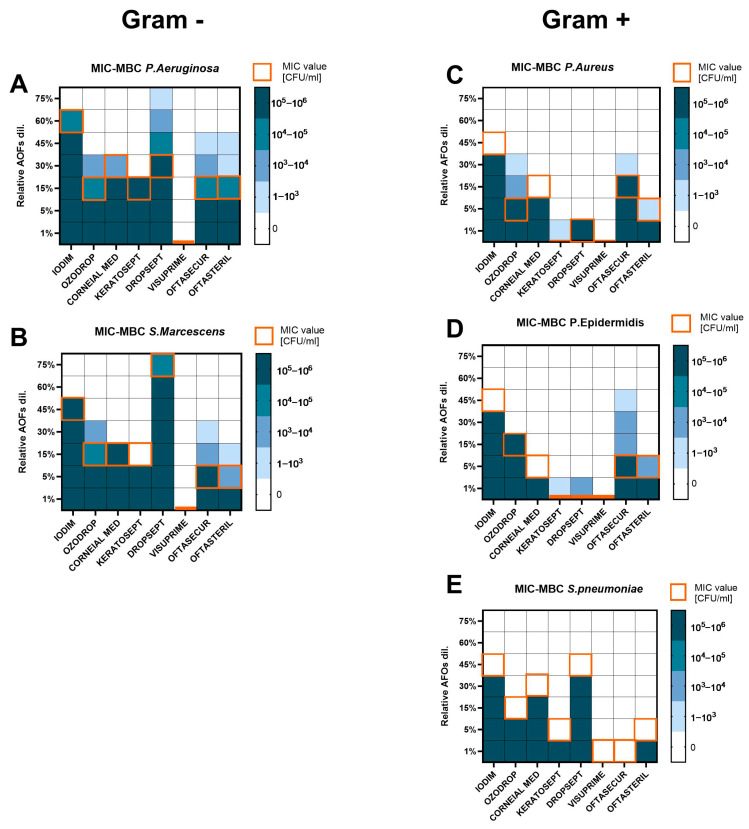
Heat map representing the MIC (orange square) and MBC (blue scale square) values for each bacterial species ((**A**,**B**) for Gram-positive; (**C**–**E**) for Gram-negative), after 24 h (for MIC) and 48 h (for MBC) of AOF exposure. Bacteria growth was expressed in CFU/mL. Assays were executed in biological triplicate and reported as mean.

To evaluate the efficacy of the AOFs against bacterial species, in vitro susceptibility tests were performed. Both Gram-negative and Gram-positive bacteria were subjected to the Minimum Inhibiting Concentration assay and Minimum Bactericidal Concentration assay. MIC results showed the highest activity of AOFs among Gram-positive compared to Gram-negative (Figure 2).

Generally, for Gram-negative bacteria, the MIC value corresponded to MBC (Figure 2A,B). Gram-negative bacteria required a higher dosage of products to be inhibited in growth or killed. *P. aeruginosa* and *S. marcescens* were completely susceptible to Visuprime (MIC and MBC = 1%). Keratosept was effective at 15%. Ozodrop, Corneal MED, Oftasecur, and Oftasteril showed MIC values in a range of 5–30% and MBC of 15–45%, with growth up to 2-fold dilution over the MIC values for Oftasecur and Oftasteril against *P. aeruginosa. S. marcescens* showed growth only at the first dilution over MIC with Ozodrop (up to 10^4^ CFU/mL), Oftasecur, and Oftasteril. Iodim was effective only at 60% and 45% on *P. aeruginosa* and *S. marcescens*, respectively. Dropsept was completely inactive against *S. marcescens* (MIC and MBC ≥ 75%), while *P. aeruginosa* seemed to be more susceptible to the same product with a MIC value of 30% but growth still present at 3-fold dilution over the MIC (10^3^ CFU/mL, MBC ≥ 75%).

Among Gram-positive bacteria, the higher MIC value did not exceed 45% of product dilution and in some cases, inhibiting and bactericidal activity was shown at the lowest tested dilution. Gram-positive bacteria showed no growth over the MIC value for the most of compounds. As reported in Figure 2C,D, *S. aureus* and *S. epidermidis* were killed by 1% dilution of Keratosept, Dropsept, and Visuprime, whereas Oftasteril and Corneial MED were active at 5–15% dilution. Iodim, Ozodrop, and Oftasecur needed to reach 30–45% dilution to be completely effective against Staphylococci. Specifically, *S. aureus* showed significant growth after the MIC only against Ozodrop with CFU/mL up to 10^4^, whereas for Keratosept, Oftasecur, and Oftasteril the growth over the MIC was up to 10^3^ CFU/mL. *S. epidermidis* showed up to 10^4^ CFU/mL growth in the presence of Oftasecur, while Keratosept, Dropsept, and Oftasteril showed fewer colonies on an agar plate, in a count up to 10^3^ CFU/mL. *S. pneumoniae* was also tested and showed the highest susceptibility to Visuprime and Oftasecur with a MIC value of 1%, as shown in Figure 2E. A dilution of 5% was active for Keratosept and Oftasteril. whereas for the other drugs, MIC values moved from 15% to 45%. No plate growth was observed over the MIC values for *S. pneumoniae*.

### 3.2. The FCM Was Useful to Measure the AOFs Activity

To deeply characterize the bacterial viability 24 h after exposure, the experimental conditions previously used for the MIC evaluation were analysed by FCM. As previously stated, the fluorescence measured with SYTO 9/PI dual staining is directly related to the integrity of the bacterial membrane. An NT and CLX control sample was included in all experiments for comparison purposes. Three distinct bacterial subpopulations were thus identified: live bacteria with intact membranes (green fluorescence), membrane-damaged “intermediates”, and dead bacteria (red fluorescence) as shown in Supplementary Figure S1.

**Figure 3:**
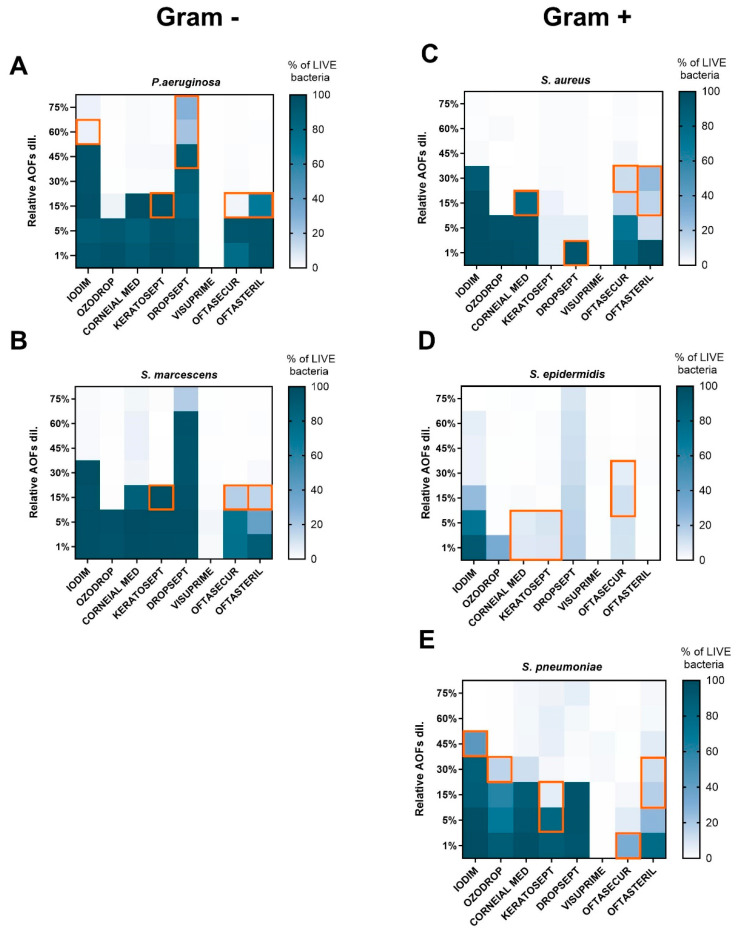
Heat map representing the relative frequency of live bacteria (blue scale square) values for each bacterial species ((**A**,**B**) for Gram-positive; (**C**–**E**) for Gram-negative), after 24 h of AOF exposure. Bacteria viability was expressed as % of green emission/total bacteria events. The MBC value forecasted by the FCM was represented by the orange squares.

The FCM results (expressed in % of live bacteria) were consistent with MIC and MBC outcomes. Concerning Gram-negative species (*P. aeruginosa* and *S. marcescens*; Figure 3A,B), it was observed that live bacteria were absent for all AOFs at minimal dilution (75–60%), with the exception of Dropsept, which presented live bacteria still at minimal dilution. When the concentration of the AOFs was reduced, it was observed that Iodim became ineffective at 30–45%, Corneial MED and Keratosept at 15%, Oftasecur and Oftasteril in the range of 5–15%, and Ozodrop at 5%. It was observed that Visuprime retained its bactericidal property even at the maximum dilution condition (1%). Also, in the case of Gram-positive species (*S. pneumoniae*, *S. aureus*, *S. epidermidis*), Visuprime retained its bactericidal activity at the maximum dilution. Generally, the Gram-positive bacteria required a lower AOF dosage to be effective against the bacteria growth.

As reported in Figure 3C,D, *S. aureus* and *S. epidermidis* were killed by 1% dilution of Keratosept and Visuprime. Iodim confirms this activity at dilutions from 75 to 45%; whereas *S. aureus* appears to be resistant at lower concentrations of Oftasecur and Iodim, corroborating the MIC and MBC results. Oftasteril affected *S. aureus* and *S. epidermidis* at very high dilutions; conversely, a high percentage of live bacteria was measured after 24 h of incubation with Iodim and Oftasecur at high dilution, in agreement with MIC–MBC measurements. Finally, evaluating the *S. pneumoniae* susceptibility (Figure 3E), we observed that Iodim, Corneial MED, and Ozodrop became ineffective when diluted at 45–30%, Dropsept and Oftasteril at 15%, and Keratosept at 5%.

To evaluate the capability of FCM to predict bacterial growth, the results obtained through the two methods were compared. As shown by the orange square in Figure 3, in some cases the FCM assay was able to predict bacterial growth 24 h in advance with respect to MBC, due to the measurement of live bacteria at the MIC concentration.

### 3.3. The TCK Classifies AOFs into Early and Late Efficacy Products

**Figure 4:**
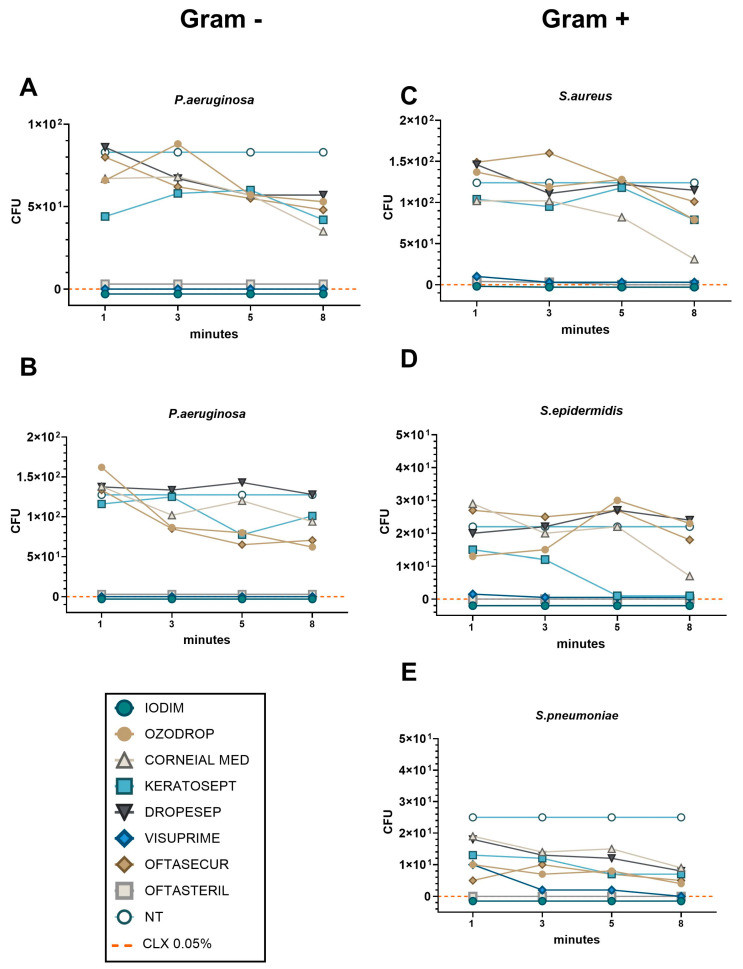
The TKC representing bacteria viability after 1–8 min of AOF exposure for each bacteria strain ((**A**,**B**) for Gram-positive; (**C**–**E**) for Gram-negative). Bacteria viability was expressed as CFU after 24 h of plate culture. NT control represented the value obtained by seeding 150 CFU of bacteria species. The efficacy control was obtained using CLX 0.05% (orange dashed line). The lines of Iodim, Oftasteril, and Visuprime are so close together that they are indistinguishable from each other, so we have nudged the lines above and below the zero line to better distinguish the trace of the three compounds.

The time-killing assays were performed for 8 min, with bacteria (150 CFU/mL) being exposed to 75% of AOF dilution. Figure 4A–E shows TKCs for all AOFs against the different microbial species.

Considering the Gram-negative species *P. aeruginosa and S. marcescens* (Figure 4A,B), we observed the absence of bacterial growth after only 1 min of exposure to the Iodim, Visuprime, and Oftasteril solutions. In contrast, all other AOFs require at least 8 min to achieve partial efficacy, without reaching the same level of efficacy as the CLX control. Similarly, when considering Gram-positive species, we measured the absence of bacterial growth after exposure to Iodim, Visuprime, and Oftasteril since the first minute of incubation, as shown in Figure 4C–E. We also observed relatively rapid efficacy (8 min) of the Corneial MED solution against *S. aureus* (Figure 4C). In the case of *S. Epidermidis*, Keratosept solution demonstrated quite rapid and effective efficacy, reducing bacterial load within 5 min of exposure, meanwhile, Corneial MED needed 8 min to become effective. Different considerations could be made describing the AOFs’ effectiveness against *S. pneumoniae.* Iodim and Oftasteril confirmed their high efficacy after only 1 min of exposure, while Visuprime needed about 3 min. All other AOFs showed a partial bactericidal activity along the entire time period, with a slight improvement at 8 min.

Therefore, we could confirm that Iodim, Visuprime, and Oftasteril demonstrated “fast” effectiveness against all bacteria, whereas Corneial MED and Keratosept were found to be more efficient against Gram-positive species. It is worth noting that some other AOFs may require additional time to achieve effectiveness.

### 3.4. A High Throughput Time-Killing Method to Classify AOFs into Early and Late Effectiveness

The experimental conditions previously employed for standard TKC evaluation were analysed also by FMR to determine the kinetic activity of AOFs (at the minimal dilution of 75%) at different time points of exposure (up to 120 min). In such instances, it may be feasible to conduct a cost-effective and efficient experiment to evaluate all bacterial species and AOFs under identical conditions). The AFU (Active Fluorescence Unit) values were compared for every point/condition to the CLX referral control (dashed orange line). A 20% deviation from the standard CLX treatment was employed as a reference point for the evaluation of the AOF efficacy (grey area). The 20% difference in efficacy from a control like chlorhexidine is often used as a benchmark when evaluating new antimicrobial or antiseptic formulations [38,39,40].

**Figure 5:**
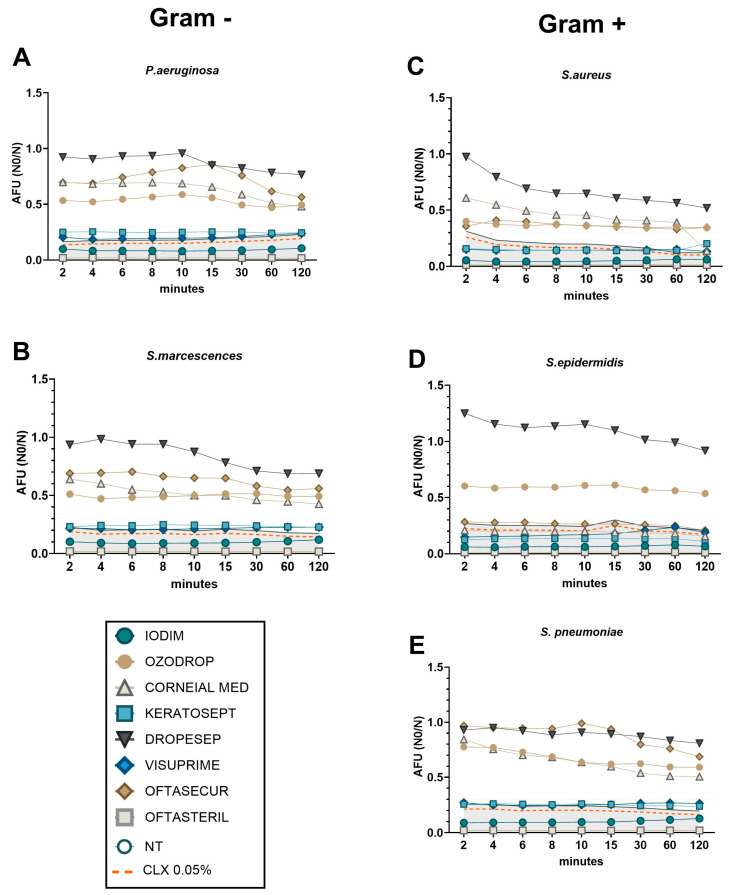
Time-killing curve representing bacteria viability after different time points of AOF exposure. Bacteria viability, of each bacteria strain ((**A**,**B**) for Gram-positive; (**C**–**E**) for Gram-negative), was measured in real time by FSP method and was expressed on AFU (N0/N). Orange dashed line represents the CLX 0.05% values that were used as efficacy control. Grey area represents the CLX 0.05% + 20% to define the efficacy of AOFs.

Consistent with the results presented in the previous paragraph, FMR experiments also demonstrated that the antibacterial efficacy of AOFs could be classified into two distinct categories based on the speed of action: early and late, in comparison with CLX referral values (Figure 5). In the context of Gram-negative species (*P. aeruginosa* and *S. marcescens*, Figure 5A,B), it was observed that Visuprime, Keratosept, Iodim, and Oftasteril, exhibited their strong antibacterial effect since the first minute of incubation, with AFU levels lower than CLX. In contrast, Ozodrop, Corneial MED, Dropsept, and Oftasecur appeared to have a partial bactericidal activity and required a minimum of 120 min to slightly reduce AFU values. Concerning the Gram-positive species (*S. pneumoniae*, *S. aureus*, and *S. epidermidis*; Figure 5C–E), we confirmed that Visuprime, Iodim, Keratosept, and Oftasteril exhibited their antibacterial effect from the first minutes of incubation, with AFU values comparable to the control CLX. Meanwhile, it was observed that Ozodrop, Oftasecur, and Corneial MED exhibited a delayed but efficacious action against *S. aureus*. Finally, the TKC obtained with *S. pneumoniae* was consistent with previous observations. The antibacterial effect of Visuprime, Iodim, Keratosept, and Oftasteril was observed from the first minute of incubation, whereas Corneial MED and Ozodrop required a longer period to become effective.

### 3.5. Tears Effect on AOFs Effectiveness

**Figure 6:**
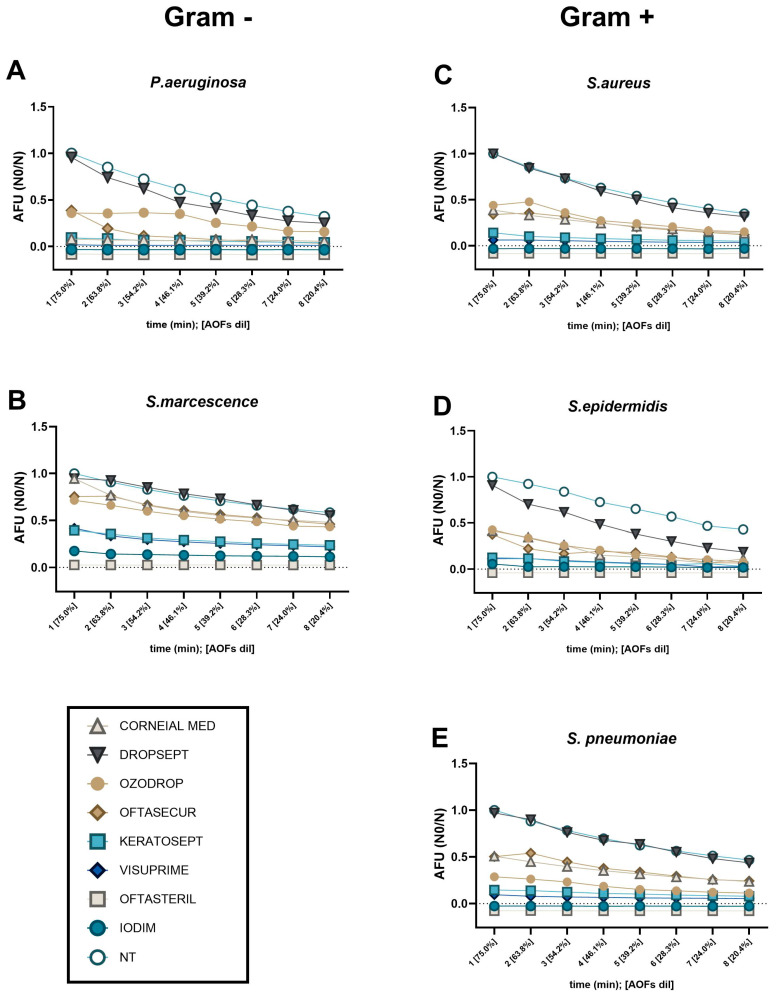
TFMC represents bacteria viability after different time points of AOF exposure. Bacteria viability, of each bacteria strain ((**A**,**B**) for Gram-positive; (**C**–**E**) for Gram-negative), was measured in real time by FMR method and was expressed as AFU (N0/N). In this kinetic test, 150 CFU of each bacteria species were exposed to the different AOFs at the minimal dilution (75%). Subsequently, every minute, we mimicked the physiological “tears dilution” by a 15% reduction of both AOFs and bacterial concentration on the ocular surface. As NT control, the fluorescence emitted by live bacteria (150 CFU) was measured being diluted by 15% every minute. In instances where AOF lines were in such close proximity that they were indistinguishable from one another, we relocated the lines below the zero line with the objective of facilitating the differentiation of the traces of the various compounds.

As previously described, the TFMC assay was applied to evaluate the “tears-dilution effect” on the AOF’s effectiveness. In the context of Gram-negative species (*P. aeruginosa* and *S. marcescens*, Figure 6A,B), it was observed that Visuprime, Keratosept, Iodim, and Oftasteril, exhibited their rapid antibacterial effect since the first minute of incubation, without being affected by tears dilution.

Considering Gram-positive species (*S. pneumoniae*, *S. aureus*, and *S. epidermidis*; Figure 6C–E), we confirmed that Visuprime, Iodim, Keratosept, and Oftasteril, exhibited their antibacterial effect from the first minute of incubation. Focusing on *S. aureus*, we observed that Ozodrop, Oftasecur, and Corneial MED, seemed to have a delayed but efficient action. Differently, Dropsept showed a late efficacy against both *S. aureus* and *S. epidermidis*. Finally, considering the TFMC obtained with *S. pneuomoniae*, we confirmed what was previously observed: Visuprime, Iodim, Keratosept, and Oftasteril exhibited their antibacterial effect from the first minute of incubation, whereas Corneial MED and Ozodrop needed more time to become effective.

## 4. Discussion

In this study, the antimicrobial activity of several AOFs was compared with different techniques for the first time. Despite Oftasteril not being an over-the-counter product, we also evaluated its efficacy, since it is frequently used as an eye disinfectant in the surgery room.

The MIC, MBC, and FMC results demonstrated that Visuprime was the only AOF highly active against both Gram-positive and Gram-negative bacteria after 24 h of incubation, while all the other products showed variable efficacy depending on the tested bacterial species. However, considering shorter times of exposure, the microbiological results obtained by the TKC assays highlighted a high antimicrobial efficacy of Visuprime, Iodim, and Oftasteril, early after a short exposure time, against all bacterial species examined in these experiments, especially Gram-negative species. The aforementioned tests allowed us to perform a classification of AOFs into two categories based on the speed and effectiveness of their action. We confirmed that Iodim, Visuprime, and Oftasteril demonstrated the most rapid efficacy against all bacteria. In contrast, Keratosept, Ozodrop, Corneial MED, Oftasecur, and Dropsept appeared to have a partial bactericidal activity at short times. Considering also the tears-dilution effect, in addition to the confirmed high efficacy of Iodim, Visuprime, and Oftasteril, Keratosept also showed rapid and stable efficacy against all bacteria, without being undermined by the effect of tear dilution. These factors are of considerable importance in the choice of AOF, due to the constant tear flow.

These observations would never have been evident considering only the results obtained with classical tests such as the MIC and MBC, which involve much longer incubation times (24–48 h) and therefore do not mimic the real situation of the ocular surface during the contact between pathogen and AOF, which consists of a few seconds. In this instance, the FCM allowed a more careful analysis of bacterial viability by separating this population into three distinct groups (viable, dead, or damaged bacteria) only after the 24-h incubation, anticipating the MBC results. Moreover, this methodology could allow a more precise estimation of the proportion of viable cells, particularly for slow-growing bacterial species such as *Streptococcus pneumoniae*, which yielded lower MBC values in comparison to MIC. The latter is based on turbidity evaluation, which can be challenging to assess visually. Furthermore, through the TKC and TFMC assay, the in vivo condition could be better replicated considering the brief contact time of AOFs with the ocular surface [16,17] and the tears dilution effects.

Previous studies conducted in this field have some important limitations. For example, some investigators performed tests by diluting 0.5 McFarland bacterial suspension in 1 mL of each AOF to obtain a final concentration of approximately 5 × 10^6^ CFU/mL. This bacterial/AOF ratio does not reflect the ocular situation at all. Moreover, the determination of the microbicidal activity was evaluated at 1, 15, 30 min; 2 h, 6 h, and overall 24 h, which didn’t resemble the real time of AOF permanence on the ocular surface or in the lacrimal sac [15]. In vitro studies have shown that a contact time between 30 s and 2 min is ideal for assessing the true efficacy of ophthalmic products [36], therefore we considered a killing curve within 8 min (to stay wide but realistic). Evaluation of longer time intervals is not useful in clinical practice.

Another important evaluation could be made considering the bacterial concentration on the ocular surface. Even when bacterial colonies are present, the number of colony-forming units (CFU) per conjunctival swab is usually much less than 100 CFU [41]. Therefore, to mimic the pre-operative prophylaxis condition, we considered 150 CFU as the referral value for the bacterial concentration [33,35].

A further crucial factor to be taken into account is that a generous amount of antiseptics is utilised in the operating room setting, with the application time extending to several minutes to ensure the efficacy of the treatment is not compromised by the dilution. In contrast, the use of antiseptic eyedrops results in a notable dilution effect. In a healthy eye, the mean lacrimal flow is 1.2 µL/min, corresponding to a turnover of 16%/min (as demonstrated in our experiments). The determination of tear volume and tear flow, as demonstrated by Mishima et al., is significantly enhanced by any stimulus. The magnitude of the stimulus directly correlates with the extent of lacrimal flow. It is evident that an antiseptic eyedrop that causes discomfort would be more diluted and therefore effective for a shorter period than one that is comfortable for the eye. In cases of conjunctivitis or keratitis, the lacrimal gland is highly efficient in producing large quantities of tears. This is an evolutionary advantage, but it also allows for the rapid removal of the disinfectant used, within seconds or minutes [30].

Therefore, considering that the AOFs are available in multi-dose or single-dose glass/plastic dropper bottles that deliver drops with a volume that ranges from 25 μL to 70 μL (average 50 μL), and starting from the evaluation performed by Shiva Kumar et al. on the low capacity of the ocular surface to contain the AOF drops [35], the reduction in AOF amount used in the tests is useful to better resemble the in vivo situation. To improve this, we introduced a new test, the TFMC, to mimic the rapid and considerable dilution of the antiseptic concentration due to the tear flow [30,31]. This test has considerable importance in the choice of AOF, due to the constant production of tears in the real situation

In accordance with the aforementioned considerations, it can be confirmed that Visuprime, Iodim, and Oftasteril have demonstrated rapid efficacy against all bacteria. Furthermore, their efficacy is not undermined by the effect of tear dilution, as the medication is very rapid and effective from the very first minute. This factor is of considerable importance in the choice of AOF, due to difficulty in keeping the eye open after applying eye drops.

The main limitation of this study is the absence of a prospective clinical evaluation to corroborate the findings. With this vision in mind, we are planning to replicate this study using ocular swab samples to closely resemble the in vivo ocular environment, with the aim of assessing the efficacy of AOF on corneal tissue using bacterial species derived from clinical isolates.

## 5. Conclusions

The main objective of our study was to support the clinician’s choice of the most suitable AOF for the prevention and treatment of ophthalmic infections. The ideal antiseptic eyedrop should be active against and high spectrum of bacterial strains, fast acting, bactericidal (non-bacteriostatic), effective in low concentration (against dilution), and non-stinging/burning (both for patient comfort and for the dilution issue). Looking at our results, we can consider Visuprime to be the most rapid and effective for ocular surface disinfection against the tested bacterial species.

Through the use of the different techniques considered here, we obtained a prompt response through a minimal number of processing steps, thereby reducing the number and cost of laboratory tests and the time required for a response. Furthermore, our proposed approach would facilitate the real-time monitoring of ocular pathogens, thereby creating an awareness of the various infectious aetiologies and a prompt evaluation of the therapy efficacy, enhancing patient care by reducing the incidence of infections and the duration of post-surgical hospitalization.

## Figures and Tables

**Table 1:** List of the ophthalmic solutions and their relative compositions.

Name	Composition
Iodim^®^	0.6% PVP-I, medium-chain triglycerides (MCTs), sodium hyaluronate, and glycerol
Ozodrop^®^	Lipozoneye (ozonated sunflower oil, soy phospholipids), hydroxypropylmethylcellulose, polyhexamethylene biguanide (PHMB), boric acid, sodium tetraborate, sodium edetate, disodium, and deionized water
Keratosept^®^	Polyvinyl alcohol 1.25%, dexpanthenol, hexamidine disethionate 0.050%, polyhexanide hydrochloride 0.0001%, methylsulfonylmethane, disodium edetate, sodium phosphate dibasic, potassium phosphate mono-basic, and purified water.
Dropsept^®^	D-alpha-tocopherol poly (ethylene glycol), 1000 succinate (Vitamin E TPGS) (0.2%), and CHX digluconate solution (0.02%)
Corneial^®^ MED	PolyhexaMethylenBiguanide: 0.0003%; cross-linked sodium hyaluronate 0.2%; hypromellose 0.2%; disodium EDTA, borate buffer, sodium chloride, excipients, and purified water.
Visuprime^®^	PQ133 100 mg, poloxamer-407 4500 mg, EDTA disodium 100 mg, and isotonic solution
Oftasecur^®^	Biosecur (2 g), hypromellose (0.15 g), phospholipids S80, boric acid, sodium tetraborate decahydrate, sodium chloride, and distilled water.
Oftasteril^®^	Iodopovidone 5% (operating room disinfectant)
Aqueous Chlorhexidine [18]	Chlorhexidine 0.05% in water

## Data Availability

The original data presented in this study will be openly available after publication in the Zenodo Repository at https://doi.org/10.5281/zenodo.13754247.

## Supplementary Materials

The following supporting information can be downloaded at: https://www.mdpi.com/article/10.3390/antibiotics13111051/s1, Figure S1: Gating strategy for the bacteria strains; Table S1: Viability Based culturable method MIC; Table S2: Viability Assessments Based on Membrane Integrity by FCM; Table S3: Viability Assessments Based on Membrane Integrity by FMR; Table S4: Viability Assessments Based on TFMC assay.
