# Supplementary material for: A New Approach to Evaluate the Bactericidal Activity of Different Antiseptic Ophthalmic Preparations Used as Surgical Prophylaxis
Source: Antibiotics (Basel). 2024 Nov 6;13(11):1051. doi: 10.3390/antibiotics13111051 (PMC11590980; doi:10.3390/antibiotics13111051)
Supplement: Supplementary file 1 [file antibiotics-13-01051-s001.zip › Supplementary Tables S1-S4.pdf]

Supplementary Table

BACTERIA STRAIN	AOFs [dil%]	IODIM	OZODROP	CORNEIAL MED	KERATO SEPT	DROPESEP	VISUPRIME	OFTASECUR	OFTASTERIL
S. marcescens	MIC median	45%	15%	15%	15%	75%	0%	15%	5%
	MIC average	50%	43%	15%	17%	75%	2%	15%	5%
	MIC SD	9%	49%	0%	13%	0%	3%	0%	0%
S. aureus	MIC median	45%	5%	15%	0%	5%	0%	15%	5%
	MIC average	40%	31%	15%	0%	5%	0%	20%	5%
	MIC SD	9%	55%	0%	0%	0%	0%	9%	0%
S. pneumoniae	MIC median	45%	15%	30%	5%	45%	1%	1%	5%
	MIC average	38%	10%	30%	3%	38%	1%	1%	5%
	MIC SD	11%	7%	0%	3%	11%	0%	0%	0%
P. aeruginosa	MIC median	60%	15%	30%	15%	30%	0%	15%	15%
	MIC average	60%	36%	30%	15%	35%	0%	15%	15%
	MIC SD	0%	43%	0%	0%	9%	0%	0%	0%
S. epidermidis	MIC median	45%	15%	5%	0%	0%	0%	5%	5%
	MIC average	45%	17%	3%	0%	0%	0%	8%	3%
	MIC SD	15%	13%	3%	0%	0%	0%	6%	3%

Sup. Table S1: Viability Based culturable method MIC. Data representing the MIC median value, average and standard deviation for each bacterial species, after 24 h (for MIC) of AOFs exposure. The values were expressed in percentage-dilution of AOFs. Assays were executed in biological triplicate.

BACTERIA STRAIN	AOFs [dil%]	IODIM	OZODROP	CORNEIAL MED	KERATO SEPT	DROPESEP	VISUPRIME	OFTASECUR	OFTASTERIL
S. marcescens	75%	1,65	0,66	2,74	0,76	18,05	0,28	0,16	0,16
	60%	1,9	0,45	4,99	0	95,62	0,63	0,12	0,64
	45%	1,95	0,12	4,79	0,05	93,53	0,18	0,12	0,24
	30%	98,1	0,14	3,42	0,14	93,97	0,55	0,1	1,87
	15%	95,97	0,37	85,91	97,67	96,02	1,3	17,46	14,78
	5%	97,56	96,1	99,38	98,3	97,99	2,95	72,89	38,68
	1%	97,72	95,33	98,85	97,38	97,44	2,06	74,4	88,7
S. aureus	75%	1,01	0,04	0,01	1,31	1,36	0	1,37	0,13
	60%	0,54	1,47	0,01	1,39	1,32	0,01	0,94	0
	45%	1,53	0	0,01	1,37	1,36	0,02	3,1	0
	30%	90,67	0,03	0,02	1,6	1,4	0,04	12,35	24,83
	15%	98,08	0,06	80,08	4,53	1,34	0,04	15,25	15,07
	5%	99,18	95,65	95,63	6,32	6,15	0,01	71,61	12,15
	1%	98,97	98,14	95,94	6,06	92,85	0,05	80,27	97,95
S. pneumoniae	75%	0	0,06	2,67	3,77	5,72	0,08	0	2,51
	60%	0	0,12	2,45	5,47	2,34	0,13	0,45	2,12
	45%	44,32	0,22	2,7	5,36	1,5	2,07	0,27	6,95
	30%	87,05	15,22	11,56	2,56	0,96	2,32	0,02	11,46
	15%	88,08	59,59	88,3	6,12	94,4	0,13	2,58	17,42
	5%	98,21	68,67	92,11	80,49	93,47	0,02	6,99	26,38
	1%	99,76	88,42	96,92	89,13	93,19	0,07	31,49	78,34
P. aeruginosa	75%	4,42	0,44	1,49	0,91	28,11	0,1	0,49	0
	60%	4,9	0,18	1,77	0,8	21,43	0,07	0,43	0,56
	45%	94,3	0,03	1,91	2,22	89,11	0,01	0,88	0,14
	30%	94,89	0,32	1,35	1,25	88,66	0,12	1,24	0,04
	15%	96,01	4,14	98,08	96,71	84,93	0,08	3,2	68,02
	5%	89,3	91,88	85,84	93,39	91,87	0,1	93,07	93,74
	1%	92,77	95,87	90,45	96,04	92,83	0,28	77,54	94,17
S. epidermidis	75%	0	0,32	0,6	0,34	9,19	0,45	0	0,45
	60%	5,84	0,38	0,29	1,19	10,78	0,39	0	0,39
	45%	5,2	0,33	0,27	0,84	11,51	0,75	0,01	0,75
	30%	5	0,32	0,24	1,18	12,33	0,85	6,13	0,85
	15%	24,66	0,53	1,7	0,38	14,56	0,31	10,9	0,31
	5%	71,6	0,56	7,01	9,59	15,52	0,28	10,69	0,28
	1%	91,99	32,02	8,16	8,58	16,32	0,25	9,92	0,25

Sup. Table S2: Viability Based on Membrane Integrity by FCM. Data represents the relative frequency of live bacteria values for each bacterial specie, after 24h of AOFs exposure. Bacteria viability was expressed as% of green emission (SYTO9)/total bacteria events (morphological gate). The stopping role set on 30.000 events on morphological gate as described in Supplementary figure 1.

BACTERIA STRAIN	Time [minutes]	IODIM	OZODROP	CORNEIAL MED	KERATO SEPT	DROPESEP	VISUPRIME	OFTASECUR	OFTASTERIL	CLX 0.05%
S. marcescens	2	0,102	0,510	0,640	0,231	0,936	0,218	0,689	0,019	0,188
	4	0,091	0,471	0,602	0,241	0,984	0,215	0,694	0,018	0,167
	6	0,086	0,483	0,549	0,239	0,940	0,204	0,703	0,018	0,171
	8	0,089	0,488	0,529	0,248	0,940	0,206	0,663	0,019	0,174
	10	0,090	0,498	0,500	0,244	0,875	0,213	0,650	0,018	0,163
	15	0,092	0,513	0,499	0,242	0,783	0,217	0,648	0,016	0,174
	30	0,098	0,515	0,460	0,238	0,710	0,223	0,581	0,018	0,164
	60	0,107	0,492	0,447	0,230	0,687	0,226	0,545	0,016	0,149
	120	0,119	0,492	0,426	0,227	0,688	0,227	0,559	0,017	0,144
S.aureus	2	0,054	0,401	0,609	0,157	0,975	0,151	0,357	0,013	0,260
	4	0,043	0,375	0,548	0,150	0,796	0,142	0,410	0,013	0,194
	6	0,043	0,362	0,494	0,141	0,693	0,144	0,397	0,012	0,179
	8	0,043	0,374	0,458	0,143	0,649	0,143	0,374	0,012	0,167
	10	0,046	0,363	0,456	0,145	0,647	0,143	0,362	0,014	0,166
	15	0,050	0,358	0,415	0,140	0,608	0,146	0,352	0,013	0,153
	30	0,054	0,345	0,408	0,138	0,587	0,146	0,344	0,014	0,135
	60	0,063	0,350	0,391	0,142	0,564	0,152	0,330	0,013	0,102
	120	0,061	0,343	0,136	0,202	0,519	0,133	0,346	0,013	0,102
S. pneumoniae	2	0,089	0,775	0,843	0,257	0,929	0,271	0,969	0,021	0,213
	4	0,092	0,771	0,755	0,261	0,950	0,248	0,950	0,021	0,213
	6	0,093	0,729	0,700	0,257	0,921	0,246	0,944	0,020	0,198
	8	0,093	0,686	0,681	0,253	0,884	0,244	0,941	0,020	0,202
	10	0,096	0,636	0,636	0,261	0,908	0,250	0,990	0,020	0,202
	15	0,096	0,619	0,597	0,255	0,893	0,253	0,937	0,019	0,196
	30	0,107	0,623	0,538	0,242	0,870	0,266	0,798	0,020	0,187
	60	0,116	0,593	0,510	0,242	0,834	0,268	0,760	0,020	0,172
	120	0,128	0,590	0,505	0,238	0,809	0,264	0,687	0,020	0,162
P. aeruginosa	2	0,098	0,534	0,697	0,250	0,924	0,204	0,695	0,018	0,137
	4	0,083	0,522	0,683	0,254	0,906	0,185	0,688	0,020	0,144
	6	0,083	0,545	0,690	0,248	0,931	0,191	0,742	0,019	0,148
	8	0,083	0,565	0,695	0,246	0,935	0,196	0,787	0,018	0,148
	10	0,080	0,587	0,686	0,249	0,957	0,196	0,825	0,019	0,150
	15	0,083	0,559	0,656	0,253	0,850	0,199	0,857	0,019	0,158
	30	0,087	0,494	0,587	0,252	0,824	0,211	0,757	0,018	0,167
	60	0,094	0,470	0,509	0,241	0,784	0,228	0,615	0,018	0,177
	120	0,105	0,494	0,479	0,244	0,766	0,237	0,563	0,017	0,192
S.epidermidis	2	0,060	0,603	0,208	0,124	1,250	0,148	0,283	0,012	0,223
	4	0,058	0,584	0,199	0,133	1,155	0,154	0,277	0,014	0,211
	6	0,062	0,595	0,205	0,133	1,123	0,157	0,279	0,013	0,209
	8	0,063	0,592	0,198	0,135	1,136	0,165	0,267	0,013	0,209
	10	0,061	0,608	0,201	0,134	1,152	0,170	0,263	0,014	0,202
	15	0,066	0,612	0,191	0,137	1,099	0,178	0,266	0,014	0,251
	30	0,071	0,569	0,184	0,133	1,016	0,208	0,258	0,013	0,204
	60	0,079	0,561	0,181	0,134	0,991	0,235	0,250	0,014	0,194
	120	0,066	0,536	0,155	0,109	0,918	0,191	0,209	0,010	0,172

Sup. Table S3: Viability Assessments Based on Membrane Integrity by FMR. Data represents the bacteria viability expressed as AFU (SYTO9) fluorescence calculated as ratio between the first time point (N0) time 0/ and the consecutive time points (N) in real-time by FSP method. The CLX 0.05% values that were used as efficacy control.

BACTERIA STRAIN	Time in minutes [AOFs dil%]	IODIM	OZODROP	CORNEIAL MED	KERATO SEPT	DROPESEP	VISUPRIME	OFTASECUR	OFTASTERIL	NT
S. marcescens	1 [75,0%]	0,175	0,714	0,945	0,395	0,946	0,416	0,755	0,027	1,000
	2 [63,8%]	0,143	0,661	0,767	0,356	0,928	0,336	0,758	0,026	0,908
	3 [54,2%]	0,138	0,597	0,659	0,314	0,853	0,297	0,666	0,026	0,830
	4 [46,1%]	0,131	0,550	0,595	0,292	0,785	0,274	0,606	0,027	0,764
	5 [39,2%]	0,125	0,513	0,551	0,276	0,732	0,256	0,563	0,027	0,707
	6 [28,3%]	0,122	0,485	0,523	0,257	0,665	0,243	0,530	0,026	0,659
	7 [24,0%]	0,119	0,439	0,499	0,246	0,608	0,232	0,490	0,026	0,619
	8 [20,4%]	0,114	0,431	0,479	0,237	0,553	0,219	0,460	0,026	0,584
S.aureus	1 [75,0%]	0,018	0,440	0,387	0,141	1,000	0,063	0,340	0,016	1,000
	2 [63,8%]	0,019	0,477	0,331	0,102	0,843	0,062	0,356	0,017	0,856
	3 [54,2%]	0,019	0,360	0,284	0,089	0,731	0,055	0,318	0,016	0,734
	4 [46,1%]	0,019	0,273	0,244	0,079	0,592	0,048	0,249	0,016	0,630
	5 [39,2%]	0,019	0,241	0,210	0,069	0,503	0,043	0,201	0,017	0,542
	6 [28,3%]	0,018	0,207	0,181	0,060	0,413	0,039	0,171	0,017	0,467
	7 [24,0%]	0,016	0,164	0,156	0,055	0,357	0,037	0,145	0,016	0,403
	8 [20,4%]	0,019	0,152	0,135	0,050	0,316	0,035	0,119	0,016	0,349
S. pneumoniae	1 [75,0%]	0,024	0,287	0,507	0,146	0,971	0,095	0,503	0,023	1,000
	2 [63,8%]	0,024	0,263	0,447	0,139	0,899	0,079	0,540	0,024	0,882
	3 [54,2%]	0,024	0,232	0,396	0,124	0,763	0,071	0,446	0,023	0,782
	4 [46,1%]	0,022	0,185	0,353	0,108	0,677	0,066	0,378	0,022	0,697
	5 [39,2%]	0,024	0,151	0,316	0,103	0,636	0,061	0,339	0,022	0,624
	6 [28,3%]	0,023	0,137	0,285	0,092	0,551	0,059	0,294	0,023	0,563
	7 [24,0%]	0,022	0,122	0,259	0,085	0,482	0,057	0,256	0,022	0,511
	8 [20,4%]	0,023	0,113	0,236	0,078	0,437	0,053	0,243	0,022	0,466
P. aeruginosa	1 [75,0%]	0,018	0,360	0,081	0,093	0,958	0,019	0,388	0,020	1,000
	2 [63,8%]	0,017	0,356	0,070	0,082	0,741	0,013	0,193	0,016	0,850
	3 [54,2%]	0,017	0,363	0,068	0,064	0,622	0,013	0,115	0,016	0,723
	4 [46,1%]	0,017	0,350	0,061	0,064	0,473	0,013	0,097	0,018	0,614
	5 [39,2%]	0,017	0,254	0,066	0,056	0,408	0,014	0,074	0,016	0,522
	6 [28,3%]	0,016	0,216	0,071	0,051	0,332	0,013	0,057	0,014	0,444
	7 [24,0%]	0,017	0,164	0,063	0,046	0,273	0,014	0,046	0,016	0,377
	8 [20,4%]	0,015	0,158	0,057	0,040	0,250	0,013	0,035	0,017	0,321
S.epidermidis	1 [75,0%]	0,057	0,425	0,413	0,125	0,908	0,114	0,375	0,010	1,000
	2 [63,8%]	0,026	0,337	0,345	0,116	0,703	0,115	0,222	0,009	0,923
	3 [54,2%]	0,027	0,257	0,262	0,084	0,617	0,093	0,168	0,009	0,838
	4 [46,1%]	0,025	0,206	0,151	0,073	0,486	0,077	0,192	0,009	0,726
	5 [39,2%]	0,025	0,156	0,131	0,057	0,381	0,064	0,180	0,009	0,651
	6 [28,3%]	0,022	0,127	0,102	0,048	0,302	0,051	0,133	0,009	0,569
	7 [24,0%]	0,017	0,103	0,065	0,061	0,230	0,023	0,071	0,009	0,467
	8 [20,4%]	0,018	0,077	0,112	0,029	0,187	0,027	0,066	0,009	0,430

Sup. Table S4: Viability Assessments Based on TFCM assay. Data represents the AFU fluorescence emission value of bacteria after different time points of AOF exposure. Bacteria viability were measured in real-time by FMR method and was expressed as AFU (N0/N). Every minute, we performed a 15% reduction of both AOFs and bacteria concentration on the ocular surface. As NT control, we measured the fluorescence emitted by live bacteria (150 CFU) being diluted by 15% every minute.
