# Supplementary figures and images for: A New Approach to Evaluate the Bactericidal Activity of Different Antiseptic Ophthalmic Preparations Used as Surgical Prophylaxis
Source: Antibiotics (Basel). 2024 Nov 6;13(11):1051. doi: 10.3390/antibiotics13111051 (PMC11590980; doi:10.3390/antibiotics13111051)

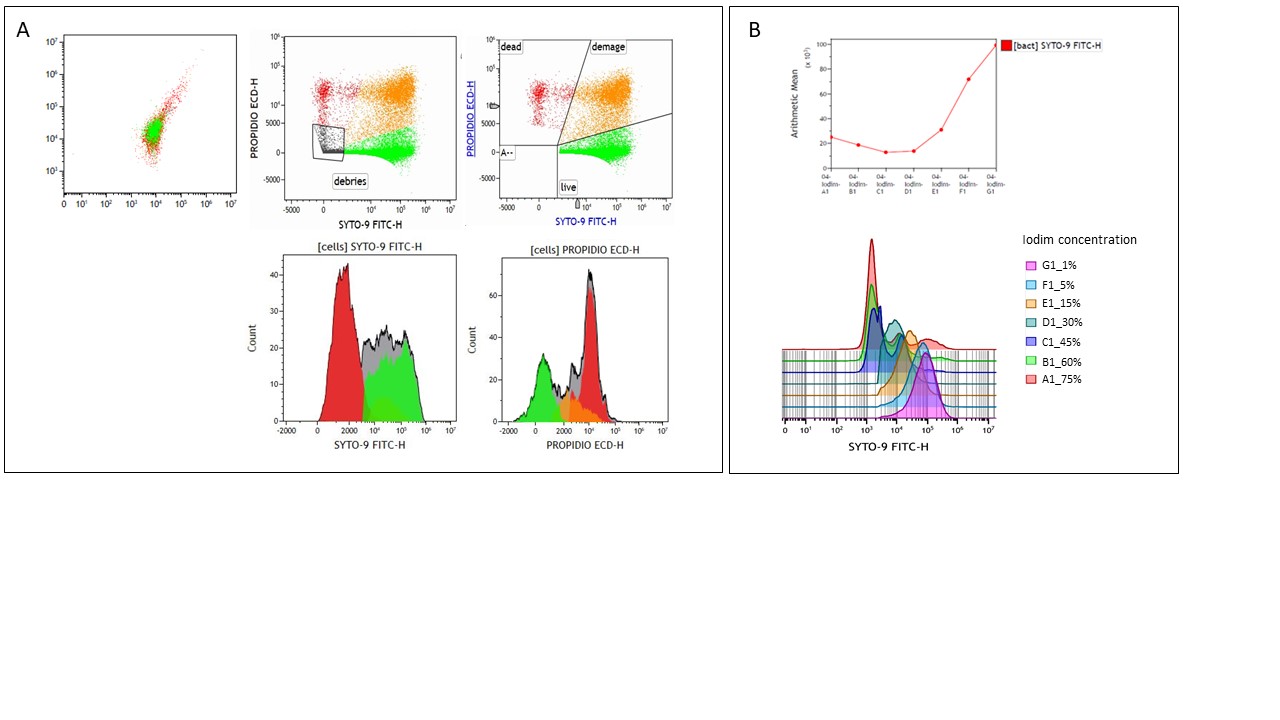

Supplement: Supplementary file 1 [file antibiotics-13-01051-s001.zip › supplementary Figure S1.jpg]
